# Low Prognostic Nutritional Index Contributes to High Adverse Events in Preeclampsia

**DOI:** 10.1155/2022/1187742

**Published:** 2022-10-11

**Authors:** Songquan Wei, Liyou Lian, Guimin Li, Jiawei Wang, Guixian Chen, Lin Yu

**Affiliations:** ^1^Department of Gynecology, The Third Affiliated Hospital of Guangzhou Medical University, Guangzhou 510000, China; ^2^Department of Internal Medicine, First Affiliated Hospital of Wenzhou Medical University, Wenzhou 325000, China; ^3^Department of Obstetrics and Gynecology, Nantong Maternity and Child Health Care Hospital Affiliated to Nantong University, Nantong, 226018 Jiangsu Province, China

## Abstract

**Background:**

Preeclampsia (PE) is a common obstetric complication that has caused significant harm to pregnant mothers. The clinical significance of poor nutritional status in PE patients is unclear. The aim of our study was to evaluate the nutritional status as measured by the prognostic nutritional index (PNI) score at admission, and its ability to predict in-hospitalization adverse events in patients with PE.

**Methods:**

We enrolled patients diagnosed with PE in the Third Affiliated Hospital of Guangzhou Medical University from January 2019 to December 2021. Patients were divided into low and high nutritional status group according to the cut-off value of PNI score at admission using the receiver operating characteristic (ROC) curve. PNI score were used to explore the relationship between PNI score and in-hospitalization adverse events presented with hazard ratio (HR) and 95% confidence intervals (CI).

**Results:**

A total of 733 patients were included in the study. The proportion of adverse events and admission to intensive care unit (ICU) was higher in the low nutritional status group than in the high nutritional status group (*P* < 0.05). ROC curve analysis revealed an area under curve (AUC) of 0.628 for PNI score and the cut-off value of PNI was 37. The free-event rates determined by KM analysis were significantly lower in the low nutritional status compared to the high nutritional status (*P* < 0.05). Adjusted multivariate analysis showed that PNI score was independently associated with favorable outcomes (HR: 2.66; 95% CI: 1.724-4.050, *P* < 0.001).

**Conclusion:**

High PNI score at admission was associated with reduced in-hospitalization risk of adverse events in patients with PE. Additional enhancing nutritional status during hospitalization may help to prevent unfavorable prognosis in clinical practices.

## 1. Introduction

Preeclampsia (PE) poses a major risk to both the mothers and their unborn fetuses. The result shows that incidence of PE morbidity is 2-8% globally [1]. PE will lead to different degrees of damage to the function of various organs including central nervous system damage, cardiovascular system damage, hepatic insufficiency, and chronic kidney diseases (CKD) [2]. Although it is known that various classical factors such as age, previous history of PE, race, pre-pregnancy body mass index (BMI), education level, multiple pregnancies, and other factors are predictive for the occurrence of PE [3], it may not always be accurately recognized due to the complex pathological process [4, 5].

Malnutrition is a common comorbidity in pregnant women and is related with worse outcomes [6]. To assess the nutritional status, BMI is calculated using the pre-pregnancy weight frequently [7], which has a fundamental role. Moreover, vitamin D level was associated with adverse pregnancy outcomes caused by PE [8]. However, the examination of nutritional status is more complex and objective. And it is unilateral and unreasonable to evaluate the nutritional status only with a single nutritional index. The prognostic nutritional index (PNI) score is composed of lymphocyte (LY) count and albumin (ALB) level, which can reflect the nutritional status of pregnant women [9]. While some studies had found the association between nutritional status and PE patients [10, 11], prognostic role of PNI in this group remains uncertain. Therefore, the aim of our study was to evaluate the nutritional status as measured by the PNI score at admission, and its ability to predict in-hospitalization adverse events in this group.

## 2. Materials and Methods

### 2.1. Clinical Data Collection

Between January 2019 and December 2021, 1171 patients diagnosed with PE in the Third Affiliated Hospital of Guangzhou Medical University were eligible in the study. According to the inclusion criteria and exclusion criteria, a total of 733 patients were included in this study finally. The inclusion criteria were as follows: (1) patients diagnosed with PE; (2) the clinical data were complete, and the follow-up data were updated. The exclusion criteria were as follows: (1) patients diagnosed with chronic hypertension with PE; (2) patients who did not deliver in our hospital; (3) patients induced labor because of fetal malformation factors; (4) patients had incomplete clinical and pathological features, such as pretreatment LY or serum albumin levels. Patients were divided into low and high nutritional status group according to the cut-off value of PNI score using the receiver operating characteristic (ROC) curve.

The significant clinical features were collected, such as age, BMI, severity of PE, admission blood pressure, gestational week of delivery, gravida, parity, adverse event, pregnancy termination way, number of admissions to intensive care unit (ICU), multiple pregnancies, laboratory metrics including white blood cell (WBC), neutrophil (NEU), LY, and ALB, echocardiograph including left ventricular ejection fraction (LVEF), left atrial diameter (LAD), and left ventricular end diastolic diameter (LVEDD), and neonatal indicator such as newborn 1 min Apgar score. Follow-up time was calculated from the date of diagnosis to the date of occurrence of adverse events including Hellp syndrome, hypertensive retinopathy, postpartum hemorrhage, placental abruption, heart failure (HF), respiratory failure, and CKD at the date.

### 2.2. Definition

The diagnosis of PE was determined according to the Practice Bulletin No. 202 American College of Obstetricians and Gynecologists [2]. PE is diagnosed in the presence of hypertension after 20 weeks of gestation with proteinuria, or in the absence of proteinuria, with impaired kidney or liver function, neurological symptoms, hematological complications, or uteroplacental dysfunction.

Other relevant definitions: (1) Hellp syndrome is a more serious form of PE, that clinical manifestations are hemolysis, elevated liver enzymes and low platelet (PLT) [2]; (2) postpartum hemorrhage is defined as: within 24 hours after the delivery, the amount of bleeding in vaginal delivery ≥500 ml, and cesarean section ≥1000 ml, and severe postpartum hemorrhage is defined as: the amount of vaginal bleeding within 24 hours after the delivery of the fetus ≥1000 ml [12]; (3) placental abruption refers to the premature separation of the placenta partially or completely before the delivery of the fetus [13]; (4) HF is a clinical syndrome characterized by a series of symptoms (dyspnea, open breathing, lower limb swelling) and signs (elevated jugular pressure, pulmonary congestion), usually caused by structural and/or functional cardiac abnormalities that lead to decreased cardiac output and/or increased intracardiac pressure [14]; (5) respiratory failure is a serious disorder of pulmonary ventilation and/or ventilation function caused by various reasons, so that effective gas exchange cannot be carried out, resulting in hypoxia with (or without) carbon dioxide retention, resulting in a series of clinical syndromes with physiological functions and metabolic disorders [15]; (6) CKD refers to the decline of renal function, which is manifested by glomerular filtration rate (GFR) lower than 60 ml/min per 1.73 m^2^, or markers of renal damage, or both, and lasts for at least 3 months, regardless of the root cause [16]; (7) Apgar score is used to evaluate the state of newborns after birth, which is acceptable and convenient. The Apgar score comprises 5 components: color, heart rate, reflexes, muscle tone, and respiration. Each of these components is assigned a score of 0, 1, or 2. The score is reported at 1 minute and 5 minutes after birth for all infants. A score of 7 to 10 as reassuring, a score of 4 to 6 as moderately abnormal, and a score of 0 to 3 as low in the term infant and late-preterm infant [17].

The PNI score was calculated with the formula: PNI score = 10 × albumin(g/L) + 0.005 × total lymphocyte count (per mm^3^) [18].

### 2.3. Statistics

For categorical variables, data were given as percentages, and for continuous variables, as mean standard deviation (SD) or median and interquartile range (IQR). Group differences were assessed by Student's *t*-tests or the Mann–Whitney *U*-tests for continuous variables, and chi-square or Fisher's exact tests for categorical data. The cut-off value of PNI score was accessed by ROC curve analysis. The event-free incidence curve was plotted via the Kaplan-Meier (KM) method. The crude or multivariate-adjusted Cox proportional regression model was used to estimate the hazard ratios (HR) and 95% confidence intervals (CI) calculated by adverse events. The statistical analyses were performed using R version 4.1.3 (The R Project for Statistical Computing, Vienna, Austria) and SPSS statistical software (SPSS statistics 26.0). Statistical tests were two-sided and *P* < 0.05 was considered statistically significant.

## 3. Results

**Figure 1:**
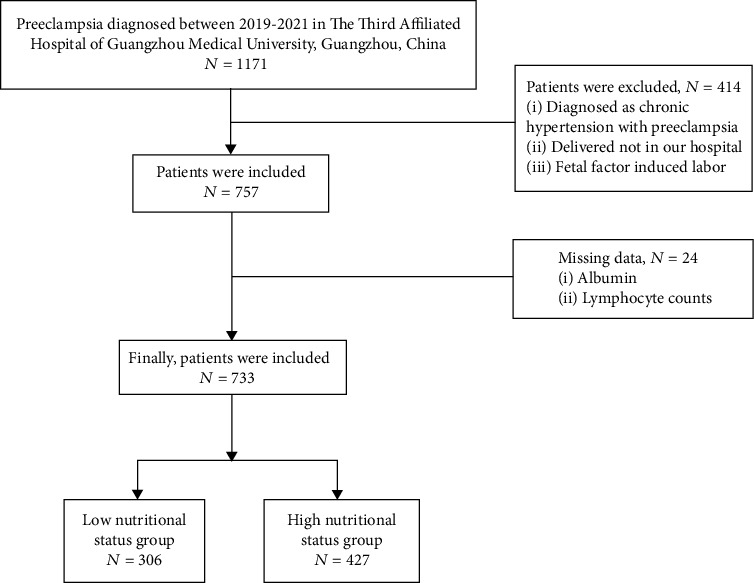
Flow chart depicting the PE patient's enrollment.

**Figure 2:**
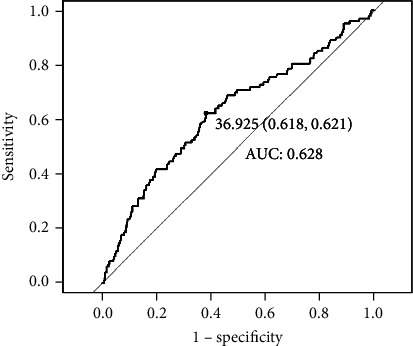
Receiver operating characteristic curve result for PNI score. Abbreviations: AUC: area under curve; PNI: prognostic nutritional index.

We enrolled 1171 women, of which 438 women were excluded from the analysis because they were diagnosed as chronic hypertension with PE, delivered not in our hospital, induced labor because of fetal factor or miss data including ALB or LY counts, leaving 733 women in the study group (Figure 1). The cut-off value of PNI score was 37. ROC curve analysis revealed an area under curve (AUC) of 0.628 (sensitivity: 62.1%, specificity: 38.2%) for PNI score (Figure 2). The baseline characteristics of the patients with low nutritional status group and high nutritional status group are summarized in Table 1. There was no significant difference between the groups in terms of age, gravida, diastolic pressure, severity of PE laboratory metrics including WBC and NEU, and echocardiograph including LVEF, LAD, and LVEDD; however, the proportion of adverse events and admission to ICU in the low nutritional status group was higher than that in the high nutritional status group. Furthermore, 18 (5.8%) women and 6 (1.4%) women in low nutritional status group and high nutritional status group developed Hellp syndrome, respectively, and placental abruption occurred in 16 (5.2%) women and 9 (2.1%) women patients, respectively. The proportions of HF and CKD in the low nutritional status group and the high nutritional status group were 7.2% vs 2.3% and 2.3% vs 0, respectively. In addition, the low nutritional status group (7.5%) was found the proportion of admission to ICU be higher than high nutritional status group (2.3%).

**Figure 3:**
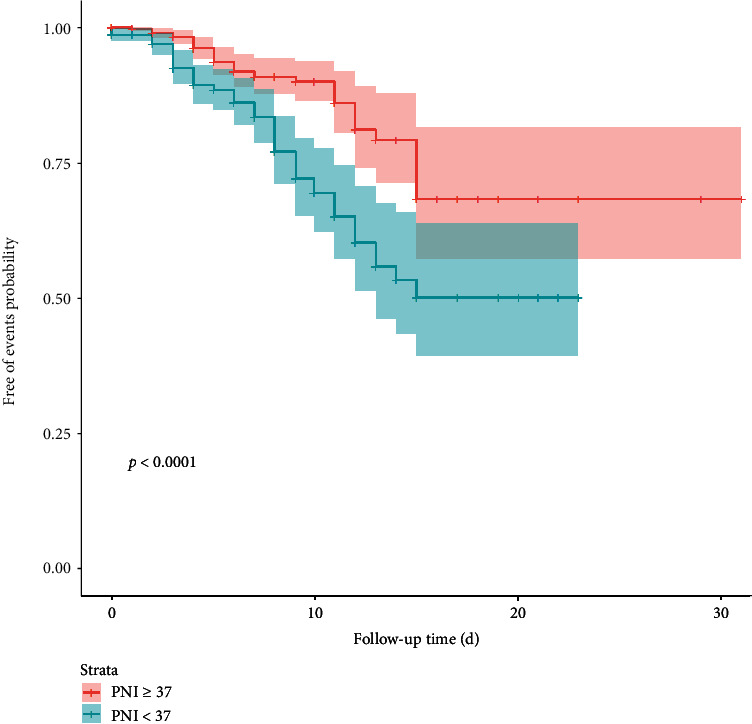
Kaplan-Meier survival probability estimates and 95% confidence intervals for patients in high and low PNI score group. Abbreviations: PNI: prognostic nutritional index.

According to the KM curves, patients in low nutritional status group had higher rates of adverse events (Figure 3). In our study, low PNI score is risk factor (HR: 2.33; 95% CI: 1.564-3.347, *P* < 0.001), and it is still meaningful to control undesirable risks (HR: 2.66, 95% CI: 1.724-4.050, *P* < 0.001) (Table 2).

## 4. Discussion

PE is one of the major causes of maternal and perinatal mortality worldwide. The presence of risk factors in patients may be related to the increased incidence of PE; however, clinical progression is not always predictable [2, 19]. Therefore, it is important for the clinician to detect prognostic factors. In our study, we examined whether nutritional status assessed by PNI score was associated with adverse outcomes in patients with PE. The ROC curve analysis indicated that PNI score predicted adverse events using a cut-off level of 37.0. Lower PNI score contributed to higher risks of adverse events in this group during the hospitalization. As far as we know, this article is the first to explore the correlation between PNI and the number of adverse events of PE patients during their stay.

The physiopathology of PE is thought to involve abnormal development of placental vasculature, with defective deep placentation and lack of transformation of the spiral arteries [20]. It has been suggested that the nutrition and the release of inflammatory factors can play an important role in placental endothelial function and oxidative stress [21–23]. In accordance with the information in the literature, malnutrition plays an important role in placental endothelial function, oxidative stress, and the expression of angiogenic factors [24]. Furthermore, malnutritional status has been associated with adverse events, including fetal growth restriction, low-birth weight, and preterm delivery [25, 26]. PNI score as an index to evaluate the nutritional status of patients is significantly associated with the prognosis of patients with gynecological cancer [27]. Zheng Feng et al. [28] and Naoko Komura et al. [29] proved that a decreased PNI score pretreatment was a poor prognostic factor in patients with ovarian cancer. PNI score, which is calculated based on the serum albumin concentration and total LY count in the peripheral blood, can reflect the nutritional status [30]. Thus, in this study, we investigated the value of PNI score in predicting adverse events during hospitalization in patients with PE before termination of pregnancy. However, we compared the difference between low nutritional status group and high nutritional status group, which suggested that the low nutritional status group had a higher proportion of admission to ICU and occurrences of adverse events including Hellp syndrome, placental abruption, and heart failure during the hospitalization. Further, it showed that lower PNI score was associated with worse clinic outcomes in PE patients. We also found that the level of ALB was significantly lower in the low nutritional status group compared with the high nutritional status group. ALB is recognized as an important marker for long-term malnutrition and systemic stress response. Low level of ALB in low nutritional status group might be attributable to reduce synthesis caused by kidney dysfunction and increased consumption due to organ damage including kidney dysfunction. Although ALB is recognized as an important marker for long-term malnutrition and systemic stress response, ALB levels are influenced by several factors [30]. So, it is necessary to evaluate the nutritional status through multiple factors. PNI is not only composed of ALB levels but also composed of LY count. It has shown inflammation including imbalance between the generation of reactive oxygen species and the antioxidant defensive system and vascular endothelial damage is related to placental dysfunction, which may lead to PE [31, 32]. It is proved that the levels of proinflammatory cytokines such as tumor necrosis factor-*α* (TNF-*α*) and interleukin-6 (IL-6) were significantly increased in PF patients (*P* = 0.0001, *P* = 0.0001) while the anti-inflammatory cytokines such as interleukin-4 (IL-4) and interleukin-10 (IL-10) were downregulated (*P* = 0.0001, *P* = 0.0001) in comparison to normal pregnancy [33]. As the results of our article show, as another important component of PNI score, the count of LY was significantly lower in low nutritional status group. Refer to previous studies the decreased LY may be considered a reflection of impaired immune function and a sharp increase in cytokines.

All in all, the PNI score is composed of Alb level and LY count, which can more comprehensively reflect the status of patients with PE. In addition, compared with other time-consuming prognostic markers, blood samples of PNI score can be easily obtained from the results of routine blood and blood biochemical, which is more convenient and inexpensive. According to the blood markers of PNI score before delivery, clinicians can more accurately evaluate the patient's condition and provide individualized treatment options.

There are several limiting points in this study. First, due to the nature of single institution research, the selection bias cannot be avoided. It is worth noting that this paper uses a dichotomous categorization using cut-off values, although this method is often done and its practice and impact are often overlooked in medical statistics texts [34]. Using arbitrary cut-off values to divide patients into two groups resulted in significant statistical bias and incomplete correction for confounders. At last, the study is retrospective, which lacks PNI score before pregnancy to predict adverse events in patients with PE during hospitalization. Hence, further prospective studies and randomized controlled trial are needed to validate the data set forth in the study.

## 5. Conclusion

As a simple and noninvasive blood indicator, PNI score can predict adverse events in patients with PE during hospitalization. Our data suggest that PE patients with low PNI score before delivery have a higher proportion of adverse events during hospitalization. The PNI score can potentially help clinicians make individualized diagnosis and treatment plans.

## Figures and Tables

**Table 1:** Baseline characteristics of enrolled patients.

Variable	Low nutritional status	High nutritional status	*P*-value
Participants (*n*, %)	306 (41.746)	427 (58.254)	
Age (y)	32 (28-36)	32 (29-35)	0.952
BMI (kg/m^2^)	21.5 (19.6-24.0)	23 (20.7-25.9)	< 0.001
Preeclampsia (*n*, %)			0.060
Mild	176 (57.5)	288 (67.4)	
Severe	130 (42.5)	139 (32.6)	
Admission blood pressure (mm Hg)			
Systolic pressure	143 (131-158)	139 (130-150)	0.008
Diastolic pressure	93 (85-102)	92 (85-99)	0.083
Gestational week of delivery	35 (32-37)	37 (34-38)	< 0.001
Gravida	2 (1-3)	2 (1-3)	0.077
Parity	1 (1-2)	1 (1-2)	0.034
Events (*n*, %)	97 (31.7)	66 (15.5)	< 0.001
Hellp syndrome	18 (5.8)	6 (1.4)	0.001
Hypertensive retinopathy	6 (2.0)	4 (0.9)	0.211
Postpartum hemorrhage	27 (8.8)	37 (8.7)	0.940
Placental abruption	16 (5.2)	9 (2.1)	0.022
Heart failure	22 (7.2)	10 (2.3)	0.002
Respiratory failure	1 (0.3)	0	0.237
Chronic kidney diseases	7 (2.3)	0	0.002
Pregnancy termination way (*n*, %)			< 0.001
Cesarean	148 (48.4)	315 (73.8)	
Eutocia	158 (51.6)	112 (26.2)	
Admission to ICU (*n*, %)	23 (7.5)	10 (2.3)	0.001
Multiple pregnancy (*n*, %)			< 0.001
Yes	101 (33.0)	81 (19.0)	
No	205 (67.0)	346 (81.0)	
1 min Apgar score	10 (8-10)	10 (9-10)	< 0.001
Follow-up time (d)	7 (5-9.25)	5 (4-9)	< 0.001
Laboratory metrics			
WBC (10^9/L)	8.96 (7.14-12.04)	9.49 (7.92-11.56)	0.056
NEU (10^9/L)	6.84 (5.04-10.04)	6.83 (5.55-8.72)	0.814
LY (10^9/L)	1.39 (1.12-1.69)	1.84 (1.51-2.24)	< 0.001
HGB (g/L)	113 (103-123)	121 (110-129)	< 0.001
PLT (10^9/L)	190 (148-234)	222 (180-268)	< 0.001
RDW (%)	14.0 (13.2-15.2)	13.8 (13.1-14.8)	0.006
ALB (g/L)	26.2 (23.3-28.4)	32.4 (30.1-34.7)	< 0.001
ALT (U/L)	12.8 (8.7-19.8)	11.3 (8.3-16.6)	0.010
AST (U/L)	19.9 (16.4-25.0)	18.2 (13.8-21.9)	0.016
BUN (*μ*mol/L)	4.71 (3.69-6.44)	4.12 (3.27-5.29)	< 0.001
UA (*μ*mol/L)	446 (373-525)	402 (323-481)	< 0.001
Cr (*μ*mol/L)	68 (56-81)	51 (58-68)	< 0.001
24 h PRO (g/24 h)	0.84 (0.39-3.01)	0.50 (0.35-1.16)	< 0.001
Echocardiograph			
LVEF (%)	65 (61-67)	66 (62-68)	0.430
LAD (mm)	35 (32-38)	35 (33-37)	0.910
LVEDD (mm)	44 (42-47)	45 (42-47)	0.297

Abbreviations: BMI: body mass index; WBC: white blood cell; NEU: neutrophil; LY: lymphocyte; HGB: hemoglobin; PLT: platelet; RDW: red blood cell distribution width; ALB: albumin; ALT: alanine aminotransferase; AST: aspartate aminotransferase; BUN: blood urea nitrogen; UA: uric acid; Cr: serum creatinine; 24 h PRO: 24-hour urinary protein; LVEF: left ventricular ejection fraction; LAD: left atrial diameter; LVEDD: left ventricular end diastolic diameter.

**Table 2:** Association between the PNI score and undesirable risk.

	HR	95% CI	*P*-value
Model 1	2.33	1.564-3.347	< 0.001
Model 2	2.36	1.573-3.578	< 0.001
Model 3	2.66	1.724-4.050	< 0.001

Model 1: unadjusted, Model 2: adjusted for age and BMI, Model 3: adjusted for age, BMI, and baseline health status (diabetes, congenital heart disease, chronic kidney disease, immune system diseases, hyperlipoidemia and liver failure). Abbreviations: BMI: body mass index; PNI: prognostic nutritional index; HR: hazard ratio; CI: confidence intervals.

## Data Availability

The data used to support the findings of this study are available from the corresponding author upon request.
